# Microbiome‐Mediated Resistance of Wild Tomato to the Invasive Insect *Prodiplosis longifila*

**DOI:** 10.1111/1758-2229.70190

**Published:** 2025-09-09

**Authors:** Stalin Sarango Flores, Viviane Cordovez, Ben O. Oyserman, Luisa M. Arias Giraldo, Nejc Stopnisek, Jos M. Raaijmakers, Pieter van ’t Hof

**Affiliations:** ^1^ Department of Microbial Ecology Netherlands Institute of Ecology Wageningen the Netherlands; ^2^ Institute of Biology Leiden University Leiden the Netherlands; ^3^ Department of Microbiological Research National Laboratory of Health, Environment and Food Maribor Slovenia; ^4^ Colegio de Ciencias Biológicas y Ambientales Universidad San Francisco de Quito Quito Ecuador; ^5^ Instituto de Microbiología, Colegio de Ciencias Biológicas y Ambientales Universidad San Francisco de Quito Quito Ecuador

**Keywords:** centre of origin, indigenous microbiome, *Prodiplosis* resistance, rhizosphere *Actinoplanes*, tomato

## Abstract

Plant roots are colonised by diverse communities of microorganisms that can affect plant growth and enhance plant resistance to (a) biotic stresses. We investigated the role of the indigenous soil microbiome in the resistance of tomato to the invasive sap‐sucking insect *Prodiplosis longifila* (Diptera: Cecidomyiidae). Native and agricultural soils were sampled from the Andes in Southern Ecuador and tested, in greenhouse bioassays, for leaf tissue damage caused by *P. longifila* on domesticated *Solanum lycopersicum* cv. Moneymaker and wild tomato *S. pimpinellifolium*. We observed no significant differences in insect damage between domesticated and wild tomatoes grown in live native or agricultural soils. However, when grown in sterilised native and agricultural soils, wild tomato was more severely affected by the insect than the domesticated tomato. Microbiome analyses revealed that soil sterilisation impacted overall rhizobacterial diversity and abundance in wild tomato. Particularly, *Actinoplanes* abundance was reduced upon sterilisation, which significantly correlated with loss of insect resistance. Metagenome analyses and genome assembly of Micromonosporaceae (*Actinoplanes* family) suggested a putative association between motility, chemotaxis, membrane transport, chorismate, and lanthipeptide biosynthesis and insect resistance. This indicates that wild *S. pimpinellifolium*, in contrast to domesticated *S. lycopersicum*, relies on specific members of the root‐associated microbiome for *P. longifila* protection.

## Graphical abstract

Soil microbiome‐mediated resistance to Prodiplosis longifila in wild tomato. Wild tomato Solanum pimpinellifolium exhibited reduced leaf damage from P. longifila when gronw in live soil compared to sterilized soil. Sterilization disrupted root‐associated microbiome, particularly reduced the abundance of Actinoplanes, which is linked to biosynthetic pathways such as chorismite and lanthipeptide production. These findings indicate that the wild tomato, in contrast to the domesticated tomato, relies on specific members of the soil microbiome to enhance resistance against P. longifila.

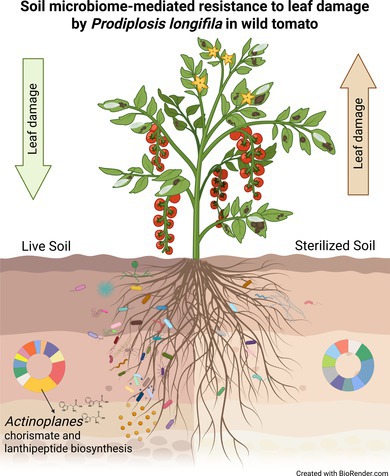

## 1 Introduction

**FIGURE 1:**
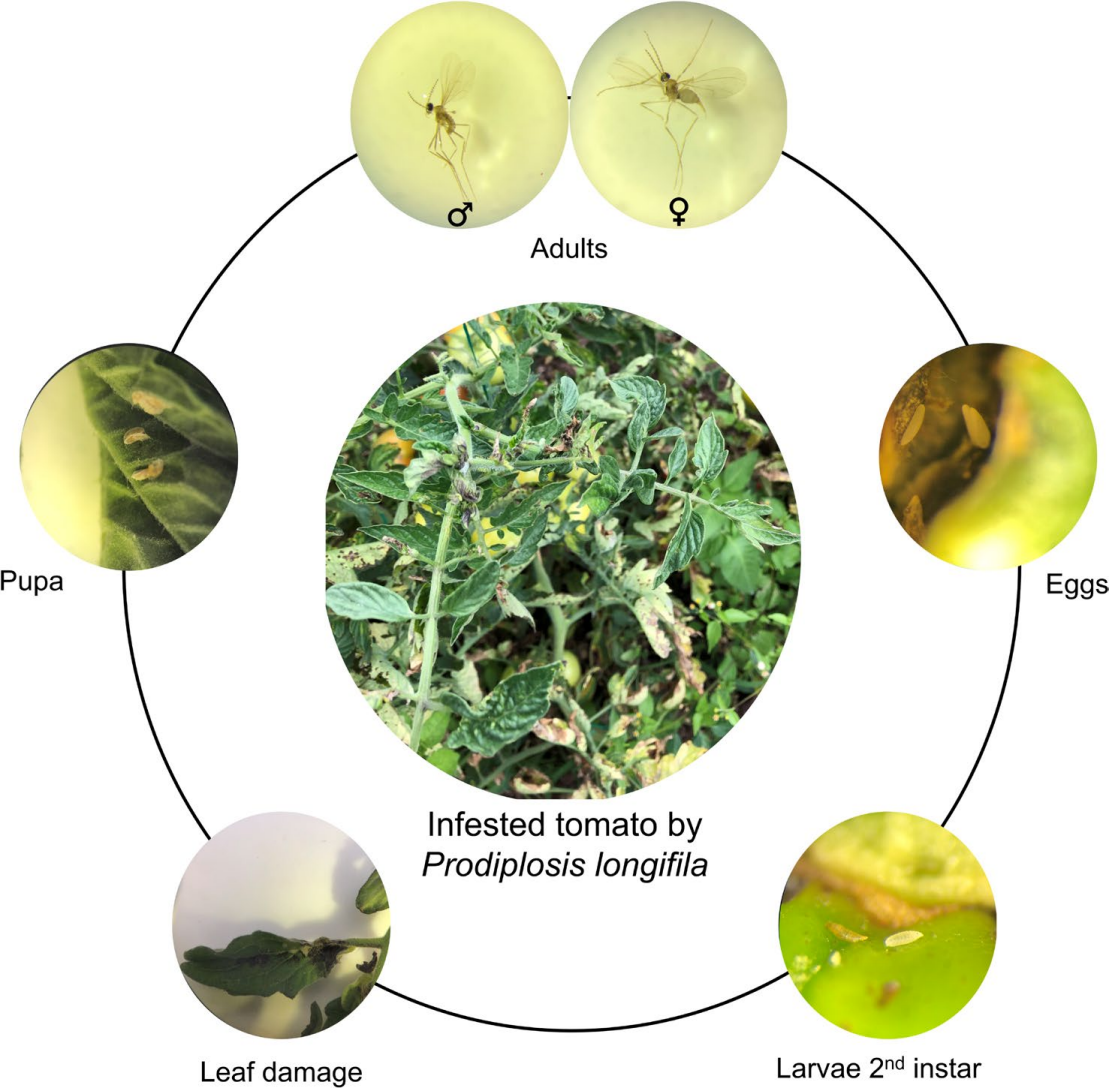
Life cycle of *Prodiplosis longifila* Gagné (Diptera: Cecidomyiidae) (photos by SSF). The centre panel shows severe damage on a tomato (*S. lycopersicum* cv. Elpida) field crop grown in Loja, Ecuador. The outer panels illustrate the insect's life cycle. Second‐instar larvae (bottom two panels) were used to infest greenhouse‐grown tomato plants, and leaf damage was assessed 7 days post‐infestation.

Root‐associated microorganisms contribute to plant growth and health via the production of hormones, enhanced nutrient acquisition, and protection from pathogens and insect pests (Carrillo et al. 2019; Choi et al. 2020; Lee et al. 2021; Meshram and Adhikari 2024; Pineda et al. 2017; Smulders et al. 2021; Tronson et al. 2022; Tronson and Enders 2025). Most of the current knowledge on how soil‐ and root‐associated microorganisms impact aboveground insect herbivory arises from single microorganisms and modern crop cultivars grown under controlled conditions in greenhouse soils (Friman et al. 2021; Rashid and Chung 2017; van de Mortel et al. 2012). However, research on how root‐associated microbiomes of wild crop relatives in native habitats impacts plant fitness, including resistance to insects, remains largely unknown. In this context, it has been postulated that wild crop relatives and their native microbiomes have co‐evolved to withstand (a) biotic stresses (Barajas et al. 2020; Pérez‐Jaramillo et al. 2016; Wallenstein 2017). In contrast, environmental disturbances within agricultural systems combined with genetic modifications by plant breeding may have led to the loss of beneficial interactions between domesticated plants and their microbiomes (Cordovez et al. 2019; O'Brien et al. 2021; Oyserman et al. 2021). To begin to understand the role of the soil and root microbiome in the protection of wild crop relatives against (a) biotic stresses, we sampled native and agricultural soils from the Andean region in Southern Ecuador and tested these soils in greenhouse bioassays with domesticated *Solanum lycopersicum* and wild tomato *S. pimpinellifolium* for leaf tissue damage caused by the insect *Prodiplosis longifila* Gagné (Diptera: Cecidomyiidae) (Figure 1). This sap‐sucking invasive insect is an economically important pest of tomato and other solanaceous crops in South America (Duque‐Gamboa et al. 2018; Hernandez et al. 2015; Valarezo Cely et al. 2003; Velasco‐Cuervo et al. 2016) and designated a priority for Pest Risk Analysis (PRA) by the EPPO Panel on Phytosanitary Measures (EPPO 2017). It can cause up to 100% loss of tomato in Colombia and up to 60% loss in Ecuador (Constante Tubay et al. 2023; Valarezo Cely et al. 2003).

To evaluate the protective potential of the tomato microbiome against this insect pest, we (1) assessed the role of the microbiome of native and agricultural soils on tomato leaf damage by *P. longifila*; (2) performed a comparative analysis of the rhizosphere microbiome composition of wild tomato *S. pimpinellifolium* and domesticated tomato *S. lycopersicum*; and (3) conducted metagenomics on soils with contrasting phenotypes to identify putative microbial traits associated with the protection of wild tomato against *P. longifila*. We found that microbiome disruption by soil sterilisation caused a significant increase in leaf damage by *P. longifila* in wild tomato grown in native and agricultural soils, but not in domesticated tomato. The taxonomic diversity of the tomato rhizosphere bacterial microbiome was different between native and agricultural soil, but no substantial differences in taxonomic composition were observed between domesticated and wild tomato. Interestingly, CCA (Canonical Correspondence Analysis) revealed that leaf damage levels (low, medium, high) significantly influenced the composition of rhizobacterial communities. Several genera, including *Actinoplanes*, showed clear associations with low–medium leaf damage levels. These findings support the hypothesis that variation in herbivory not only alters microbial community composition but may also select specific rhizobacteria associated with plant defence. Subsequent metagenome analyses pinpointed putative traits of *Actinoplanes*, including motility, chemotaxis, membrane transport, and secondary metabolism, that may contribute to enhanced insect resistance in wild tomato.

## 2 Materials and Methods

### 2.1 Soil Sampling and Processing

Agricultural and native soils were collected in Zapotillo (Loja province, Ecuador). Agricultural soil was collected from a recently harvested corn farm close to the Catamayo river (El Coco site, 4°22′44.0″S, 80°14′11.8″W), while native soil was collected from the right bank of the Alamor river (La Ceiba site, 4°18′07.6″S, 80°13′16.7″W) from a patch with natural vegetation mainly consisting of: *Prosopis juliflora* (Fabaceae), *Croton wagneri* (Euphorbiaceae), *Ipomoea purpurea* (Convolvulaceae), *Cromolaena* sp. (Asteraceae), *Parthenium hysterophorus* (Asteraceae), *Hyptis* sp. (Lamiaceae), and wild tomato *S. pimpinellifolium* (Solanaceae). Soils were transported to the greenhouse of the National University of Loja, air‐dried for 7 days at room temperature, sieved (2‐mm‐diameter mesh) to remove stones and plant debris, and stored at room temperature in the shade until further processing.

### 2.2 Experimental Design

Domesticated tomato *Solanum lycopersicum* cv. Moneymaker and its close wild relative *S. pimpinellifolium* were grown in native and agricultural soils. In order to enrich the soil with rhizosphere microorganisms, live soils passed a ‘microbial activation’ phase, which consisted of growing either wild or domesticated genotypes in the agricultural and native soils for 30 days prior to the experiment. Afterwards, the complete plants and roots were removed from each pot, and half of the soil volume was subjected to heat sterilisation (1.5 atm and 121°C, 1 h, for two consecutive times with 24 h in between) whereas the other half of the activated soil remained untreated. To ensure consistency, we used the same tomato genotypes for microbiome activation as those assigned to each corresponding treatment in the experiment. Tomato seeds were surface sterilised with 5 mL of ethanol 80% (v/v) and vortexed for 2 min. Then, the ethanol was removed and 5 mL of sodium hypochlorite 1.5% (v/v) was added, vortexed for 10 min and discarded. Finally, five washing cycles were done with 5mL volumes of sterile demi water, vortexing for 2 min and removal of the water. The seeds were then placed on a wet filter paper in a Petri dish containing 5 mL of sterile demi water and incubated at 25°C for 2 days. Before sowing the seeds, each soil type was moistened by adding 10% (v/w) of water and distributed in pots (350 g per pot). In each pot, three pre‐germinated seeds (radicle length ~1 cm) were sown at 2‐cm depth and after the seedlings emerged, two were removed to obtain one single seedling per pot. This allowed us to have seedlings with uniform sizes across the different treatments. The experiment consisted of 2 tomato genotypes × 2 soil types (agricultural, native) × 2 soil conditions (live, sterilised) × 5 replicates, each subjected to either larvae‐infested or uninfested conditions, for a total of 80 experimental units, which were organised in a completely randomised design. Considering the differences in growth between domesticated and wild tomatoes, watering was done with tap water on a total pot weight basis. Plants were grown for 34 days under greenhouse conditions (temperature day/night: 25/22°C; relative humidity 60%; 12 h natural light) until the 4th true‐leaf developmental stage.

### 2.3 Insect Infestation

The insect *Prodiplosis longifila* Gagné (Diptera: Cecidomyiidae) was used to infest tomato leaves. Infested leaves with *P. longifila* larvae were collected from a tomato field (*S. lycopersicum* cv. Elpida) at the fruiting stage in Catamayo (Loja, Ecuador, 3°54′51.4″S, 79°19′45.8″W) and transported in a cooler to the greenhouse facilities at the National University of Loja (Ecuador). A total of 10 s‐instar larvae were carefully transferred to the upper leaves of 34‐day‐old plants using a fine paintbrush, ensuring at least one larva per leaf (Supplementary Figure S1). Each infested plant was examined for insect damage at 7 dpi (days post‐infestation). To this end, plants were harvested 1 week after infestation (41 days after sowing) by removing the entire plant from the pot. The shoots were cut at the root collar region; the leaves were detached and spread on paper and photographed with a ruler for scaling and assessing insect damage. The short 7‐day duration of the experiment was chosen based on the second larval developmental stage (L2) of *P. longifila*, which is responsible for most of the feeding damage and lasts approximately 1 week. During this stage, the larvae are sap‐suckers, inserting their mouthparts into leaf veins and feeding locally with minimal movement, typically only a few millimetres. Due to the larvae's highly localised and sedentary feeding behaviour, cages or confinement systems were not necessary. The subsequent larval stage (L3) results in the larvae falling off the plant and burrowing into the soil to pupate.

Leaf damage by *P. longifila* larvae was quantified with ImageJ (Schindelin et al. 2015). This software associates the number of pixels in the pictures with the real scale or ruler to measure the area (mm scale was used). Total leaf area and leaf damage area were determined for each leaf (Supplementary Figure S2). Leaf damage proportions were calculated by dividing the damaged area (necrosis) by the total leaf area. These data were subjected to analysis of variance (ANOVA) with a linear model using the function *lm* in RStudio environment (R 4.3.1) (R Core Team 2023). To account for unequal sample sizes in the leaf damage proportion analysis, we calculated the number of leaves per treatment and fitted the linear model incorporating weights based on leaf count (weights = 1/Leaf_count). This approach ensured that each observation contributed proportionally to the analysis, preventing treatments with fewer leaves from exerting disproportionate influence on the results. The packages dplyr (Wickham et al. 2023) and ggplot2 (Wickham 2016) were used for data structuring and plotting.

### 2.4 DNA Extraction From the Tomato Rhizosphere for Microbiome Profiling

Following the harvest of the 41‐day‐old tomato plants, roots were shaken vigorously to remove loosely attached soil. The root system was then placed into a 15 mL tube with 4 mL of LifeGuard Soil Preservation Solution and stored at 4°C. In the Laboratory of Biotechnology, National University of Loja, the rhizosphere samples were split into three subsamples with 1 g of soil and 1 mL of LifeGuard Soil Preservation Solution and placed into a 2 mL microtube and stored at −20°C until DNA extraction. To extract rhizosphere DNA, the 2mL microtubes were centrifuged at 10,000 × *g* for 1 min, and the supernatant of LifeGuard Soil Preservation Solution was discarded. The Qiagen DNeasy PowerSoil Kit (Qiagen, USA) was used to isolate the genomic DNA of the remaining soil pellet according to the manufacturer's kit protocol. DNA samples were sent to Genome Québec (Canada) for amplicon library preparation and subsequent sequencing of the V3‐V4 regions of the 16S rRNA gene using the universal bacterial primers 341F (CCTACGGGNGGCWGCAG) and 805R (GACTACHVGGGTATCTAATCC). Paired‐end sequence reads (300 bp length) were generated using the Illumina NovaSeqX Plus platform. Additionally, shotgun sequencing was performed on eight pooled rhizosphere samples: DNA of five replicates from each treatment was pooled and 20 μL (concentration 20 ng/μL) aliquots were lyophilized and subjected to library preparation and shotgun sequencing to provide paired‐end reads of 150 bp by Illumina NovaSeq 6000.

### 2.5 Amplicon Data Analysis

The compressed sequence reads contained in FASTQ format files were processed by the DADA2 v1.16.0 pipeline (Callahan et al. 2016) in the RStudio environment (RStudio Team 2020) to obtain the ASV abundance and taxonomy tables. The modelling of error rates associated with the sequencing process was adjusted to appropriately process NovaSeq data using the DADA2 pipeline, as previously suggested by Holland‐Moritz et al. (2021). The SILVA 16S ribosomal RNA gene reference database (v138) (Quast et al., Quast et al. 2013) was used for bacterial taxonomy assignment. The statistical analyses were performed in the RStudio environment and *R* software version 4.3.1 (R Core Team 2023). Packages such as vegan (Oksanen et al. 2020), phyloseq (McMurdie and Holmes 2013), metagenomeSeq (Paulson et al. 2013) and ggplot2 (Wickham 2016) were used for alpha diversity (ANOVA, Tukey HSD post hoc test), beta diversity (Bray–Curtis distance, PERMANOVA with 9999 permutations), and differential abundance analyses, while the tidyverse package (Wickham et al. 2019) was used for formatting and visualisation. The abundance data were normalised by CSS (Cumulative Sum Scaling) before further analysis. Principal Coordinates Analysis (PCoA) was done using the *cmdscale* function from the vegan package and the Bray–Curtis distance calculated previously.

Differential abundance analysis was performed using the metagenomeSeq *R* package. Microbiome data, represented by ASV (Amplicon Sequence Variant) abundances, were normalised using CSS normalisation. Low‐abundance ASVs were filtered out based on the effective sample size, determined using the *calculateEffectiveSamples* function. The model “~Treatment” along with several contrasts between treatments were applied to examine differential abundances across soil types, tomato genotypes, and soil conditions, using the *fitZig* function. Differentially abundant ASVs with significant log_2_ fold changes (adjusted *p* < 0.05) were selected for further analysis. The taxonomy associated with these ASVs was incorporated to facilitate biological interpretation and visualisation. Since soil type (agricultural vs. native) and soil condition (live vs. sterilised) were identified as the interacting factors with the most substantial effect (Supplementary Table S6) on microbial composition, ASVs that showed significant differences in abundance were grouped into distinct subsets based on combinations of soil type, tomato genotype, and soil condition. These subsets were used for comparative analysis.

To analyse the relationship between the abundance of bacterial genera and leaf damage, a Canonical Correspondence Analysis (CCA) was performed using the *cca* function from the vegan *R* package. Abundance data corresponded to ASVs differentially abundant between soil conditions (live and sterilised), identified separately for each combination of soil type (agricultural and native) and tomato genotype (domesticated and wild). Leaf damage proportions were binned into three categories (low, medium, high) using quantiles to ensure approximately equal group sizes. This was done using the *cut* function in *R*, with breaks set at the 0th, 33rd, and 66th percentiles. Leaf damage categories were then used as the explanatory variable to assess how leaf damage levels influence the distribution of bacterial genera across treatments.

### 2.6 Metagenome Data Analysis

The compressed paired‐end FASTQ files were processed using SqueezeMeta v1.5.1 (Tamames and Puente‐Sánchez 2019). Co‐assembly was done using Megahit (Li et al. 2015). 16S RNAs were predicted with Barrnap (Seemann 2014) and taxonomically classified using the RDP classifier (Wang et al. 2007). While tRNA/tmRNA sequences were predicted using ARAGORN (Laslett and Canback 2004). ORFs were predicted using Prodigal (Hyatt et al. 2010). Similarity searches for GenBank (Clark et al. 2016), EggNOG (Huerta‐Cepas et al. 2016), KEGG (Kanehisa and Goto 2000) were done using Diamond (Buchfink et al. 2015). Read mapping against contigs was performed using Bowtie2 (Langmead and Salzberg 2012) and binning was done using MaxBin2 (Wu et al. 2016) and Metabat2 (Kang et al. 2019). Results from both binning approaches were combined using DAS Tool to obtain refined bins (Sieber et al. 2018). Relevant information from the metagenomics data (ORF, contig and bin annotations, aggregated taxonomic and functional features) was exported into tables by running the SqueezeMeta utility script *sqm2tables.py* (Tamames and Puente‐Sánchez 2019) to facilitate the metadata handling for further analysis.

Bin completeness and contamination were computed using CheckM (Parks et al. 2015). Based on this, and a quality threshold choosing only bins with completeness higher than 70% and contamination less than 10%, 69 bins were selected (Supplementary Table S7). A high‐quality bin of unclassified Micromonosporaceae was submitted to RAST server (Rapid Annotation using Subsystems Technology) (Aziz et al. 2008) for functional annotation. Also, the bin file was submitted to antiSMASH software (Antibiotics & Secondary Metabolite Analysis Shell) version 7 (Blin et al. 2023) to annotate BGCs (biosynthetic gene clusters) involved in secondary metabolite production.

## 3 Results

### 3.1 Impact of Tomato Genotype and Soil Condition on Insect Leaf Damage

**FIGURE 2:**
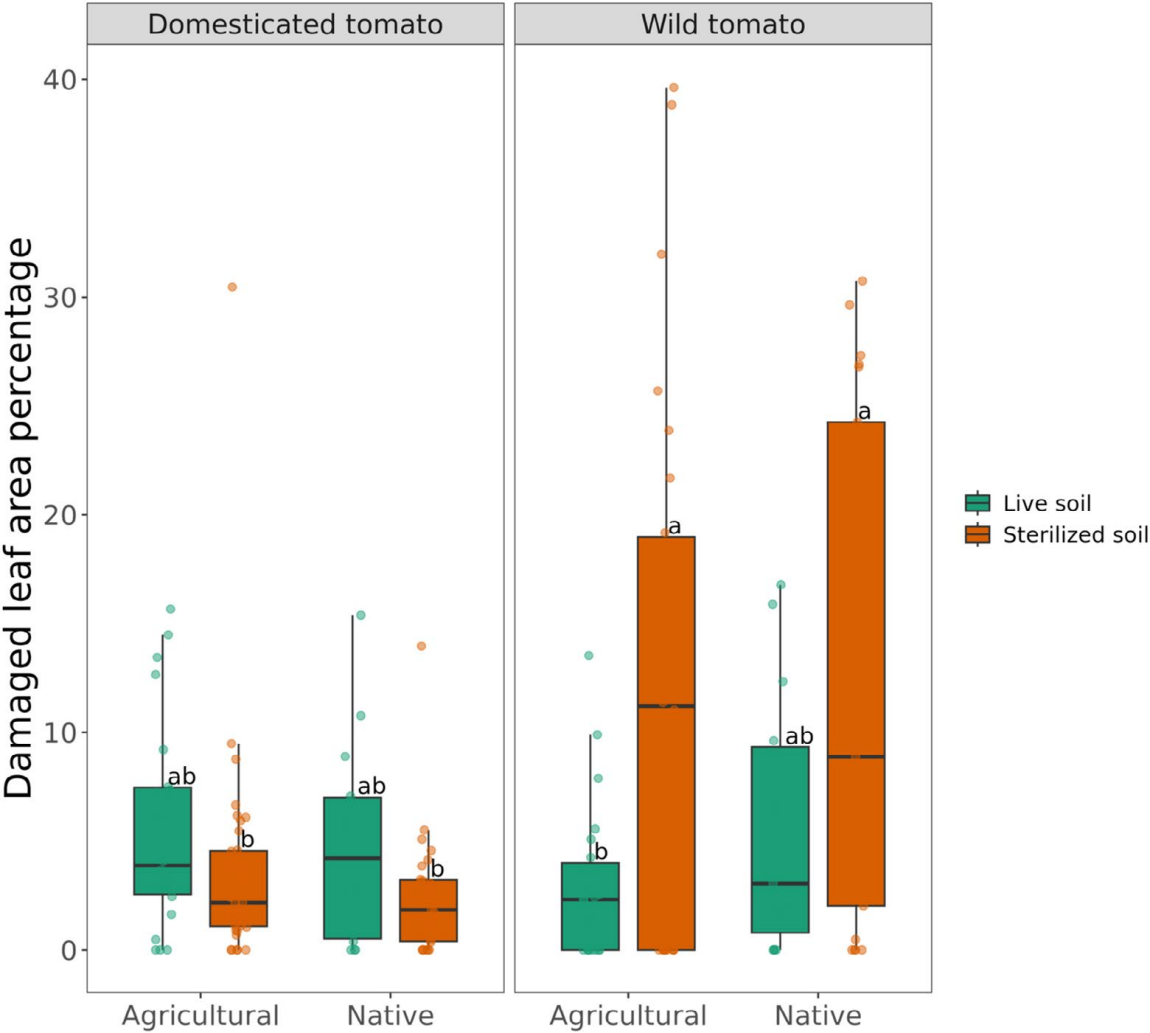
Leaf damage by *Prodiplosis longifila* on domesticated tomato *S. lycopersicum* cv. Moneymaker and wild tomato *S. pimpinellifolium* grown for 41 days in live and sterilised native and agricultural soils. Bars represent the standard deviations of the mean of damaged leaf area of five replicates. Significant differences in the percentage of insect leaf damage between the domesticated and wild tomato are indicated by different letters (Tukey HSD post hoc test *p* < 0.05).

Insect larvae caused significant leaf damage among the treatments. A linear model used to assess the effects of soil type, soil condition, and tomato genotype, along with their interactions with leaf damage proportion, explained 21.4% of the variance (adjusted *R* ^2^ = 0.182, F(7, 175) = 6.786, *p* = 3.962e‐07). The interaction between soil condition and tomato genotype had a significant effect on leaf damage proportion (*p* = 0,000226), with wild tomato grown in sterilised soils exhibiting significantly higher damage than expected from additive effects alone (Supplementary Table S3). ANOVA results confirmed that tomato genotype (*p* = 1.747e‐05), soil condition (*p* = 0.037), and their interaction (*p* = 8.278e‐06) were significant predictors of herbivory (Supplementary Table S4). Both the domesticated tomato *S. lycopersicum* cv. Moneymaker and wild tomato *S. pimpinellifolium* showed low levels of insect leaf damage when grown in agricultural (5.66% and 2.99%, respectively) or native soils (4.82% and 5.69%, respectively) (Figure 2). When the agricultural and native soils were sterilised, the domesticated tomato cultivar remained resistant to insect leaf damage. In contrast, significantly more insect leaf damage was observed when the wild tomato was grown in sterilised agricultural and native soils, compared with the respective live soils (Figure 2). Insect leaf damage on wild tomato increased to an average of 11.8% and 12.5% in agricultural and native soils, respectively. In contrast, no significant change was observed for insect leaf damage on the modern tomato cultivar grown in sterilised agricultural and native soils (3.65% and 2.45%, respectively). These results indicate that the wild tomato, in contrast to the domesticated tomato, relies on the soil (micro)biome for protection against *P. longifila*.

### 3.2 Effect of Soil Sterilisation on Tomato Rhizosphere Microbiome Composition

**FIGURE 3:**
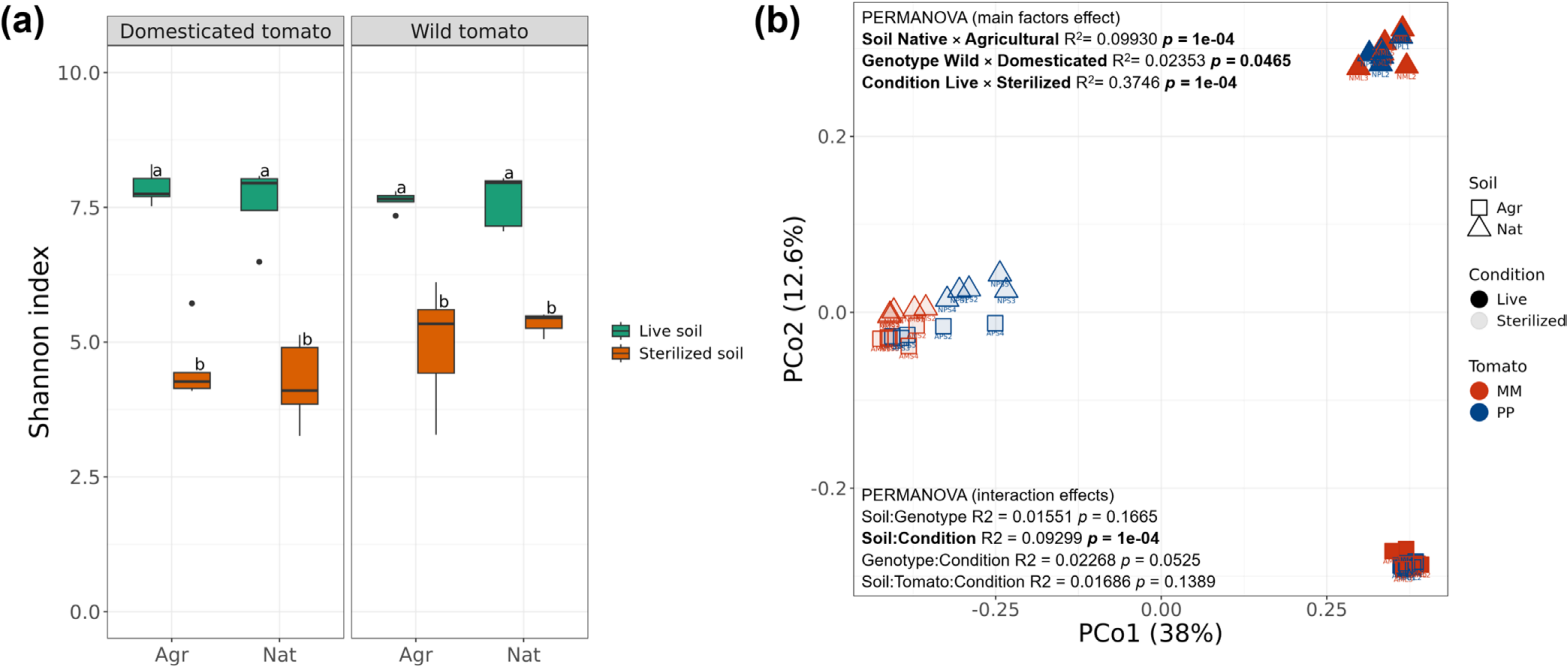
(a) Shannon diversity index of bacterial communities in the rhizosphere of domesticated *S. lycopersicum* cv. Moneymaker and of wild tomato *S. pimpinellifolium* grown in live and sterilised native and agricultural soils. Boxplots represent the mean of five replicates. Significant differences between soils and tomato genotypes are indicated by different letters (Tukey HSD post hoc test *p* < 0.05); (b) PCoA of bacterial rhizosphere of domesticated *S. lycopersicum* cv. Moneymaker (MM) and wild *S. pimpinellifolium* (PP) tomato infested by *Prodiplosis longifila* in different soil types (Agr: Agricultural; Nat: Native) and soil conditions (Live; Sterilised). PERMANOVA of main factors and interaction effects are shown.

Alpha diversity of rhizosphere bacterial communities was significantly higher for tomato plants grown in the live soil as compared to plants grown in the sterilised soils (ANOVA, *p* = 9.9e‐13). This was reflected in higher Shannon indices for tomato plants grown in live soils as compared to sterilised soils (Figure 3a). Infestation with larvae of *Prodiplosis longifila* did not significantly affect the total leaf area (ANOVA, *p* = 0.2623; Supplementary Tables S1 and S2) nor did it significantly alter the overall rhizobacterial community composition (PERMANOVA, *p* = 0.0531; Supplementary Table S5). However, significant differences in beta diversity were found between live and sterilised soil conditions (PERMANOVA, *p* = 0.0001; Supplementary Table S5 and Figure S3). When considering only the infested treatments, the interaction between soil type and condition remained significant (*p* = 0.0001), which indicated that the effect of soil type on the microbial community is influenced by soil sterilisation (Supplementary Table S6). Furthermore, sterilisation appeared to reduce the compositional differences between agricultural and native soils, as shown in the PCoA plot (Figure 3b), where sterilised samples clustered closely together regardless of soil type. This suggests that removing the native microbial community through sterilisation may homogenise rhizosphere conditions, diminishing the distinct signatures of each soil type.

### 3.3 Changes in Wild Tomato Rhizosphere Composition by Soil Sterilisation Are Associated With Leaf Damage

**FIGURE 4:**
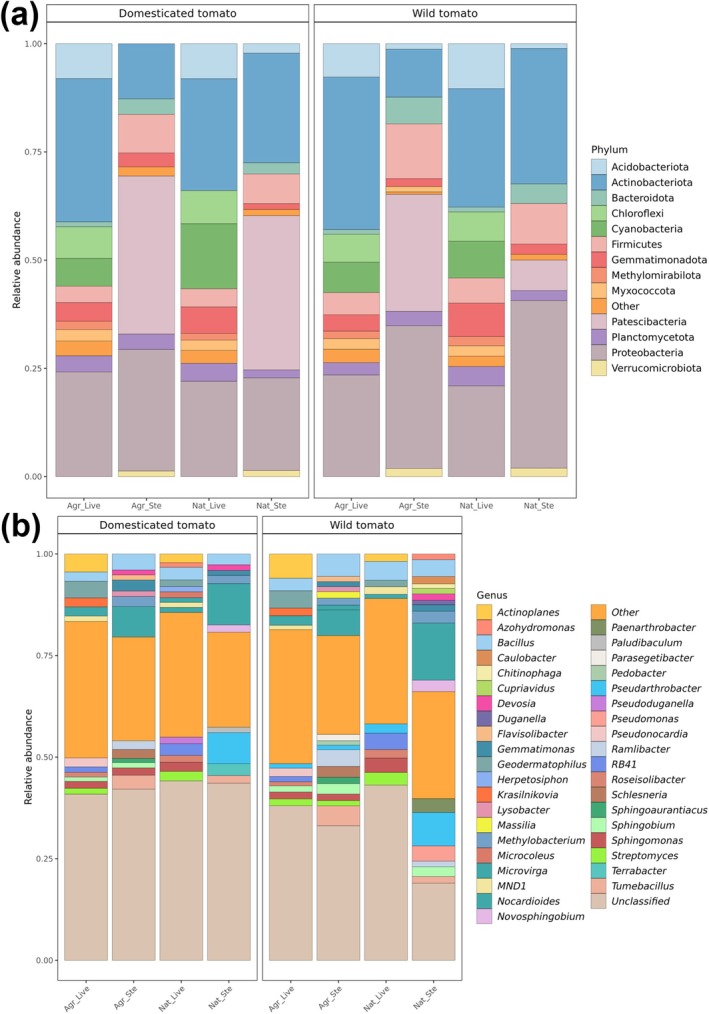
Composition of bacterial phyla (a) and genera (b) in the rhizosphere of domesticated *S. lycopersicum* cv. Moneymaker and wild tomato *S. pimpinellifolium* in live (Live) and sterilised (Ste) soil conditions; significant differential ASVs among live and sterilised agricultural (Agr) and native (Nat) soils were grouped according to their phylum or genus and plotted as stacked bar charts; “Other” category corresponds to grouped phyla or genera with relative abundance < 0.01.

**FIGURE 5:**
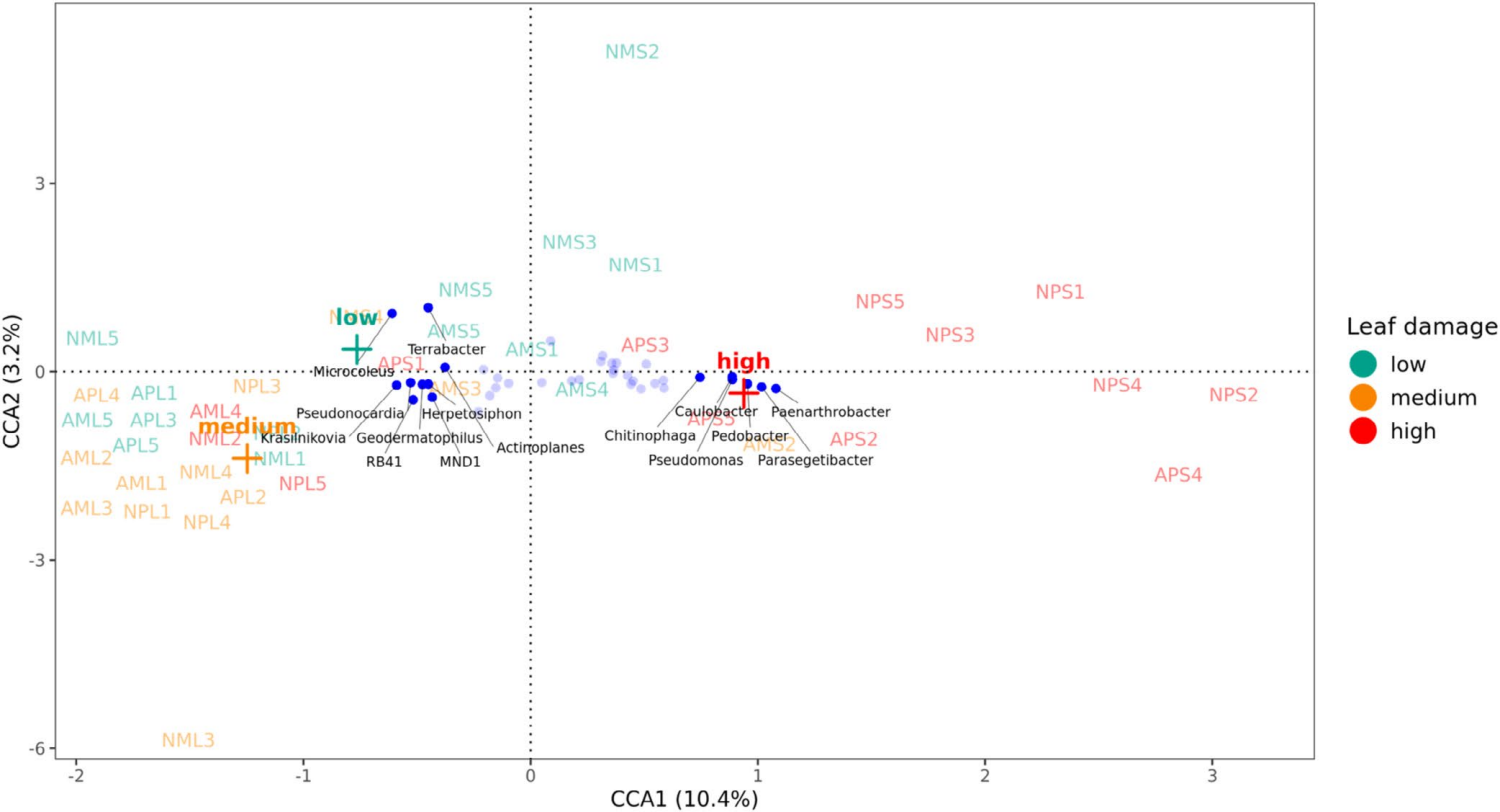
Canonical Correspondence Analysis (CCA) ordination plot showing the distribution of bacterial community composition and its relationship with treatments and leaf damage levels caused by *Prodiplosis longifila* larvae on tomato. Centroids (+) represent leaf damage levels, and sample names are coloured according to the corresponding damage category. *Actinoplanes*, *Geodermatophilus*, *Microcoleus*, *Terrabacter*, *Krasilnikovia*, *Pseudonocardia*, *MND1*, *RB41*, and *Herpetosiphon* are shown as more strongly associated with low to medium leaf damage levels, while *Paenarthrobacter*, *Pseudomonas*, *Parasegetibacter*, *Pedobacter*, *Caulobacter*, *and Chitinophaga* were associated with high leaf damage levels. Sample codes with A: Agricultural or N: Native soil; M: Domesticated *S. lycopersicum* cv. Moneymaker or P: Wild tomato *S. pimpinellifolium*; L: Live or S: Sterilised soil condition.

To explore whether shifts in the rhizobacterial community composition are associated with the level of leaf damage caused by *P. longifila*, amplicon data of the wild tomato grown in live or sterilised native and agricultural soils were subjected to differential abundance analysis. This analysis showed that 260 ASVs were shared between live agricultural and native soils, whereas 101 ASVs were shared between sterilised agricultural and native soils (Supplementary Figure S4). We observed an impact of soil sterilisation on the relative abundance of specific members of the phyla Actinobacteriota, Acidobacteriota, Chloroflexi, Cyanobacteria, Gemmatimonadota and Myxococcota (Figure 4a). More specifically, CCA identified genera such as *Actinoplanes*, *Geodermatophilus*, *Microcoleus*, *Terrabacter*, *Krasilnikovia, Pseudonocardia*, *MND1*, *RB41*, and *Herpetosiphon* as being more strongly associated with the bacterial communities of live soils, while *Paenarthrobacter*, *Pseudomonas*, *Parasegetibacter*, *Pedobacter*, *Caulobacter*, and *Chitinophaga* were key contributors to the variation observed in sterilised soils (Figure 4b and Figure 5). The CCA results suggest that leaf damage levels significantly influence the bacterial community (CCA model ANOVA, *p* = 0.007), with the first constrained axis (CCA1) explaining a substantial portion of the variance (10.4%).

### 3.4 Functional Features of Key Rhizobacterial Genera Associated With Leaf Damage

**FIGURE 6:**
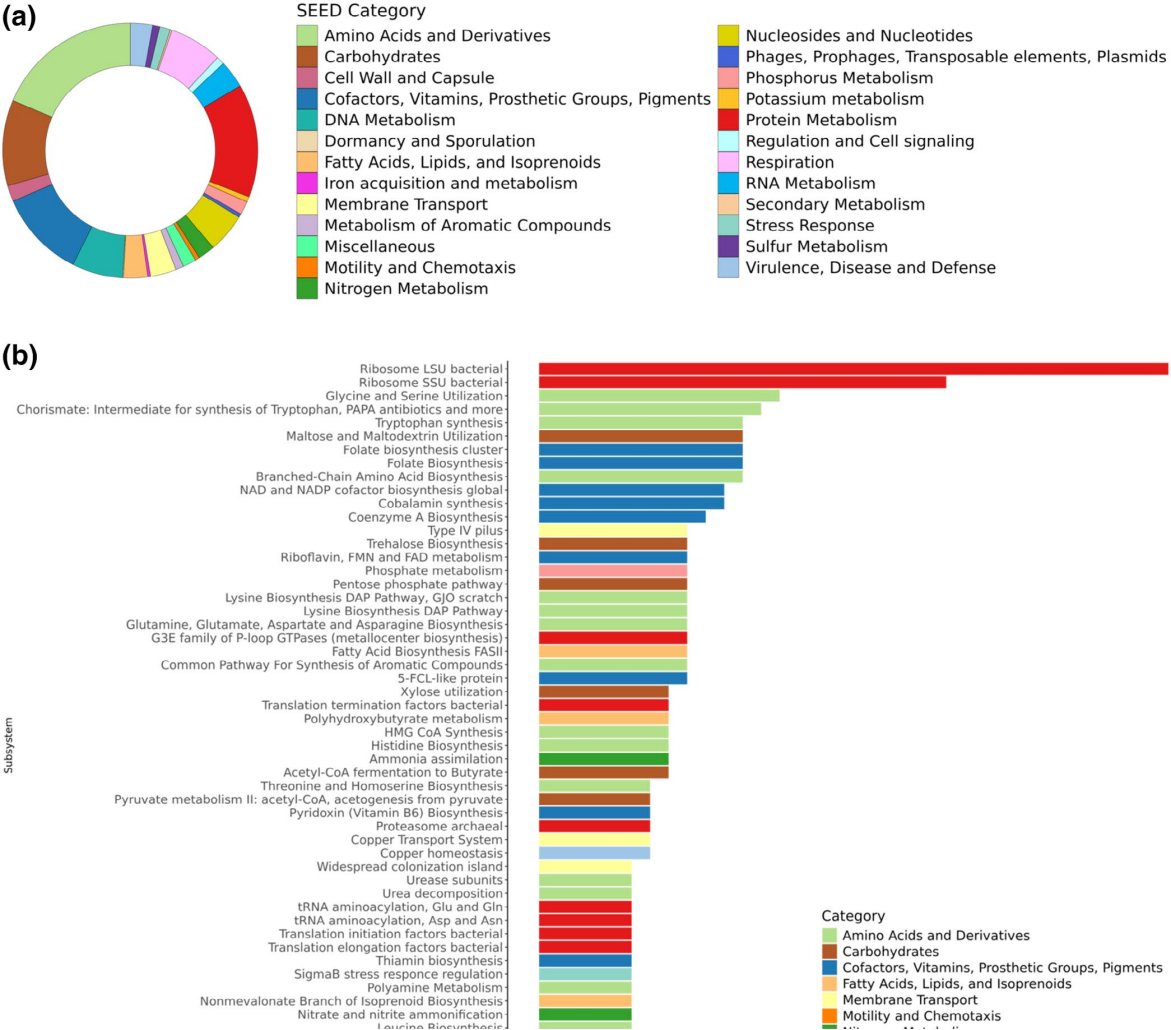
(a) Relative abundance of SEED Categories annotated by RAST (Rapid Annotation using Subsystems Technology) of MAG *Actinoplanes* bin 580 from the rhizosphere of wild tomato *S. pimpinellifolium* that showed a significantly association with low‐medium leaf damage by *Prodiplosis longifila*; the relative abundance of protein‐encoding genes was grouped in their respective SEED Categories and plotted as donut charts; (b) Number of protein‐encoding genes of main SEED Subsystems in MAG *Actinoplanes* bin 580.

To gain insight into the functional traits of the bacterial genus *Actinoplanes*, which was found to be associated with low and medium levels of leaf damage, we performed shotgun sequencing with a pooled set of rhizosphere soil samples (five replicates of each treatment, each consisting of eight pooled samples). A high‐quality metagenome‐assembled genome (MAG), bin 580, with 88.42% completeness and 2.59% contamination, was classified at the family level as Micromonosporaceae, which includes the genus *Actinoplanes* (Supplementary Table S7). To investigate their functional traits, the MAG was annotated using RAST and antiSMASH. *Actinoplanes* bin 580 contained 883 protein‐encoding genes and was characterised by features related to motility and chemotaxis, as well as secondary metabolism, according to SEED categories. Moreover, this MAG exhibited a higher number of genes associated with membrane transport, nitrogen metabolism, protein metabolism, secondary metabolism, sulfur metabolism, and virulence, disease and defence (Figure 6; Supplementary Table S8).

A total of 11 biosynthetic gene clusters (BGCs) were identified in *Actinoplanes* bin 580. Among these, three were annotated as: a type III polyketide synthase (PKS) cluster predicted to encode loseolamycins A1 and A2; an aminopolycarboxylic‐acid BGC associated with the siderophore ethylenediaminesuccinic acid hydroxyarginine (EDHA); and a terpene BGC predicted to encode isorenieratene, although with low similarity to known references (Table 1; Supplementary Figure S5).

**TABLE 1:** Biosynthetic gene clusters (BGCs) predicted by antiSMASH bioinformatics tool from bins of Micromonosporaceae bins 580 and *Nocardioides* bin 2.78 enriched in the rhizosphere of the wild tomato *S. pimpinellifolium* infested by *Prodiplosis longifila* in live and sterilised soil, respectively.

Bin ID	Length (nt)	BGC type	Secondary metabolite	Similarity	Reference in MiBIG database	Organism in MiBIG database
*Actinoplanes* bin 580	23,195	T3PKS	Loseolamycin A1, Loseolamycin A2	64%	BGC0002362	*Micromonospora endolithica*
*Actinoplanes* bin 580	13,418	Aminopolycarboxylic‐acid	EDHA	88%	BGC0002568	*Streptomyces scabiei* 87.22
*Actinoplanes* bin 580	10,650	Terpene	Isorenieratene	25%	BGC0000664	*Streptomyces griseus* subsp. *griseus* NBRC 13350

## 4 Discussion

It has been postulated that wild crop relatives and their native microbiomes have co‐evolved to withstand (a) biotic stresses (Barajas et al. 2020; Pérez‐Jaramillo et al. 2016; Wallenstein 2017). In contrast, environmental disturbances within agricultural systems combined with genetic modifications by plant breeding may have led to the loss of beneficial interactions between domesticated plants and their microbiota (Cordovez et al. 2019; O'Brien et al. 2021; Oyserman et al. 2021). In our study, we observed no significant differences in leaf damage by the sap‐sucking invasive insect *P. longifila* on both domesticated and wild tomatoes grown in live native or agricultural soils (Figure 2). However, microbiome disruption by soil sterilisation caused a significant increase of insect leaf damage in wild tomato grown in native and agricultural soils, but not in domesticated tomato. These results suggest that wild tomato *S. pimpinellifolium*, in contrast to domesticated *S. lycopersicum*, relies on specific members of the root‐associated microbiome for protection against the invasive insect *P. longifila* aboveground. It also suggests that the domesticated tomato has an intrinsic resistance to the invasive insect that operates largely independently from the soil microbiome. A previous study by Carrillo et al. (2019) found that tomato's resistance to herbivory by *Manduca sexta* (Lepidoptera: Sphingidae) was not dependent on tomato domestication or soil type. However, they observed that the wild tomato *S. pimpinellifolium*, grown consecutively in the same soil, that is, ‘tomato soil’, attracted more parasitoid *Cotesia congregata* (Hymenoptera: Braconidae) compared to the domesticated tomato *S. lycopersicum*. In our experimental design under controlled greenhouse conditions, the attraction of parasitoids did not play a role. Most likely, insect resistance in our experiments is due to root colonisation and priming the plants' defence mechanisms (Pieterse et al. 2014) to resist insect attack.

Subsequent analyses showed that heat sterilisation of the soils led to a significant increase in leaf damage of wild tomato when grown in both agricultural and native soils (Figure 2). Soil sterilisation significantly reduced the alpha‐diversity of the bacterial community in the rhizosphere of tomato grown in native and agricultural soils (Figure 3a). Furthermore, the differences observed in the rhizosphere microbiome composition were primarily due to the interaction effect of soil type (agricultural, native) and soil condition (live, sterilised), rather than the tomato genotype (Figure 3b; Supplementary Table S4). This is consistent with previous studies indicating that soil exerts a stronger effect on rhizosphere assembly than plant host genotype (Cheng et al. 2020; French et al. 2020; Kandasamy et al. 2021; Smulders et al. 2021). Although rhizosphere microbiome assembly was similar for both tomato genotypes, the microbiome changes in the rhizosphere of wild tomato grown in sterilised soil suggested a putative role of the rhizosphere microbiome to minimise leaf damage by *P. longifila* (Supplementary Table S3). A comparable effect was found in *S. pimpinellifolium* infested by the aphid *Macrosiphum euphorbiae*, whose degree of infestation was influenced by the soil microbiome. Specifically, higher microbial diversity reduced aphid infestation (French et al. 2021). Moreover, the negative impact on insect performance or less damage on plants grown in an intact native microbiome has been previously described by Badri et al. (2013) for *Arabidopsis thaliana* and by Hubbard et al. (2019) for the crucifer *Boechera stricta*.

Differential abundance analyses further revealed that the abundance of several bacterial phyla was affected by soil sterilisation, such as Actinobacteriota, Acidobacteriota, Chloroflexi, Cyanobacteria, Gemmatimonadota and Myxococcota (Figure 4a). Members of the phyla Actinobacteriota and Cyanobacteria have been investigated for the production of insecticidal compounds (Berry et al. 2008; Kaur et al. 2014; Nassar et al. 1999; Sharanappa et al. 2023; Silva et al. 2022). More specifically, *Streptomyces* spp. have been reported as a source of insecticides with potent larvicidal activity (Amelia‐Yap et al. 2022, 2023; Balakrishnan et al. 2017). Here we found *Actinoplanes*, *Geodermatophilus*, *Microcoleus*, *Terrabacter*, *Krasilnikovia*, *Pseudonocardia*, *MND1*, *RB41*, *and Herpetosiphon* to be more abundant in the rhizosphere of wild tomato grown in live soil, while *Paenarthrobacter*, *Pseudomonas*, *Parasegetibacter*, *Pedobacter*, *Caulobacter*, and *Chitinophaga* were more abundant in the rhizosphere of tomato grown in sterilised soil (Figure 4b). These bacterial genera showed a significant association with the level of leaf damage caused by *P. longifila* (Figure 5). *Actinoplanes* spp. have been studied for their potential as insecticidal agents against other dipteran species such as *Drosophila melanogaster* (Diptera: Drosophilidae) (Al‐Kaabi 2004) and Aedes aegypti*i* (Diptera: Culicidae) (Balakrishnan et al. 2017; Hozzein 2017). Nevertheless, these studies primarily focused on the activity of these microbial compounds applied directly onto the insects, rather than harnessing the ability of specific microorganisms to activate plant defence mechanisms (Choi et al. 2020; Lee et al. 2021; Ling et al. 2022; Pineda et al. 2017, 2020). Additional experiments, involving isolation of *Actinoplanes* and other bacterial candidates, insect bioassays, and mechanistic analyses, will be needed to validate the putative roles of these potentially rhizobacteria in the protection of wild tomato against *P. longifila*. Extensive attempts to isolate *Actinoplanes* from wild tomato rhizosphere have not yet been successful. While plants possess both constitutive (baseline) and induced defence mechanisms, here we specifically examined microbe‐associated composition that promotes plant resistance upon herbivory stress, rather than constitutively expressed traits. In this context, the potential role of root‐associated bacteria in mediating or enhancing induced responses remains to be experimentally validated.

Nevertheless, our analysis of the MAG *Actinoplanes* bin 580 did provide some first insights into the functional features that may be associated with wild tomato's resistance to *P. longifila* (Figure 6; Supplementary Table S3). Among these, motility and chemotaxis SEED subsystem was characterised by flagellar functions such as genes encoding synthesis of peptidoglycan (FtsI) and flagellin (FlaA, FlgB, FlgC and FliE). These genes are involved in activating bacterial division and assembling the protein components of the flagellum (Jang et al. 2016; Nedeljković et al. 2021; Tsang and Bernhardt 2015). Flagella are required for bacterial motility and colonisation and adherence to host surfaces (Guerry 2007; Palleroni 1976; Uchida et al. 2011). Flagella and pili are distinctive features of *Actinoplanes* zoospores (Kimura et al. 2019; Uchida et al. 2011). Particularly, pilus genes found in ‘bin 580’, such as PilB, PilC, PilM, and PilT, may allow *Actinoplanes* zoospores to colonise and adhere to the tomato root surface (Kimura et al. 2019). Complementarily, within *Actinoplanes* bin 580, genes of the widespread colonisation island subsystem included tight adherence (TAD) genes, such as TadA, TadB, TadC, and TadZ, and the pilin‐encoding gene Flp, suggest *Actinoplanes'* capacity to colonise host tissues and surfaces of several environmental habitats (Planet et al. 2003). Furthermore, motility features have been described to be related to bacterial niche differentiation in high carbon availability as the rhizosphere (Ramoneda et al. 2024; Wu et al. 2023). Moreover, the presence of genes encoding lanthionine synthetases (LanB, LanL) of the secondary metabolism SEED category suggests the potential for the biosynthesis of lanthipeptides, which have demonstrated antimicrobial activity against Gram‐positive bacteria by disrupting cell wall biosynthesis (Li et al. 2021; van Staden et al. 2021) as well as immunomodulatory activity (Ramírez‐Rendón et al. 2023). Additionally, lanthipeptides can exert a regulatory role for aerial hyphae formation with limited antimicrobial activity in some actinobacteria (Holtsmark et al. 2008; Kodani et al. 2005). Furthermore, genes encoding amino acids and derivatives in ‘bin 580’ suggest the biosynthesis of chorismate, which is a precursor of several secondary metabolites such as aromatic compounds, including tryptophan, involved in electron transport, signalling communication, plant defence, and wound response (Corpas et al. 2021; Macheroux et al. 1999; Mishra and Baek 2021). In particular, features of the biosynthesis of chorismate and aromatic amino acids may modulate plant defence response against *P. longifila*, which has been demonstrated in other studies on induced resistance against bacterial pathogens and phloem‐feeding insects (Assis et al. 2021; Blundell et al. 2020; Shi et al. 2016). Last but not least, we also detected a putative cluster for the biosynthesis of the terpene isorenieratene, which is characteristic for certain, Actinobacteria to cope with oxidative stress (Benaud et al. 2021; Krügel et al. 1999). The antiSMASH results also suggest that *Actinoplanes* harbours various underexploited natural products. These findings further emphasise the importance of isolating *Actinoplanes* spp., testing their biosynthetic potential, and determining the putative role of these metabolites in the protection of wild tomato against *P. longifila*.

In conclusion, the results from this study highlight the potential of exploring centre‐of‐origin soils that harbour viable populations of close relatives of economically important modern crops. Such soils may serve as valuable microbial reservoirs of resistance to various pests and diseases. This is a promising area of research, particularly in understanding how belowground microbial communities, in conjunction with specific plant genotypes, can potentially promote resistance to aboveground insect pests. Therefore, the integration of genomic approaches, involving the validation of key microbial taxa, along with the assessments of plant metabolites and defence hormones, will be essential to establish and validate the mechanistic links between microbial communities and plant defence pathways. Clarifying these dynamics and the modes of action of these ‘ancestral microbiota’ (Raaijmakers and Kiers 2022) could pave the way for designing innovative agricultural practices that harness natural soil microbiomes to improve crop resilience.

## Author Contributions

Stalin Sarango Flores: methodology, investigation, data curation; writing – original draft, review and editing; Viviane Cordovez: writing – review and editing; data curation; Ben O. Oyserman: investigation, data analysis; writing – review and editing; LMAG: data curation; visualization; review and editing; Nejc Stopnisek: data curation; Jos M. Raaijmakers: conceptualization; supervision; funding acquisition, writing – review and editing; Pieter van 't Hof: supervision; review and editing. All authors contributed critically to the drafts and gave final approval for publication.

## Conflicts of Interest

The authors declare no conflicts of interest.

## Supporting information


**Data S1:** Supplementary Figures.

**Data S2:** Supplementary Tables.

## Data Availability

Raw 16S and shotgun metagenomics sequences were submitted to the European Nucleotide Archive (ENA) under the project accession number PRJEB82448.
