# Supplementary material for: Microbiome‐Mediated Resistance of Wild Tomato to the Invasive Insect Prodiplosis longifila
Source: Environ Microbiol Rep. 2025 Sep 9;17(5):e70190. doi: 10.1111/1758-2229.70190 (PMC12420677; doi:10.1111/1758-2229.70190)
Supplement: Supplementary file 1 — Data S1: Supplementary Figures. [file EMI4-17-e70190-s002.docx]

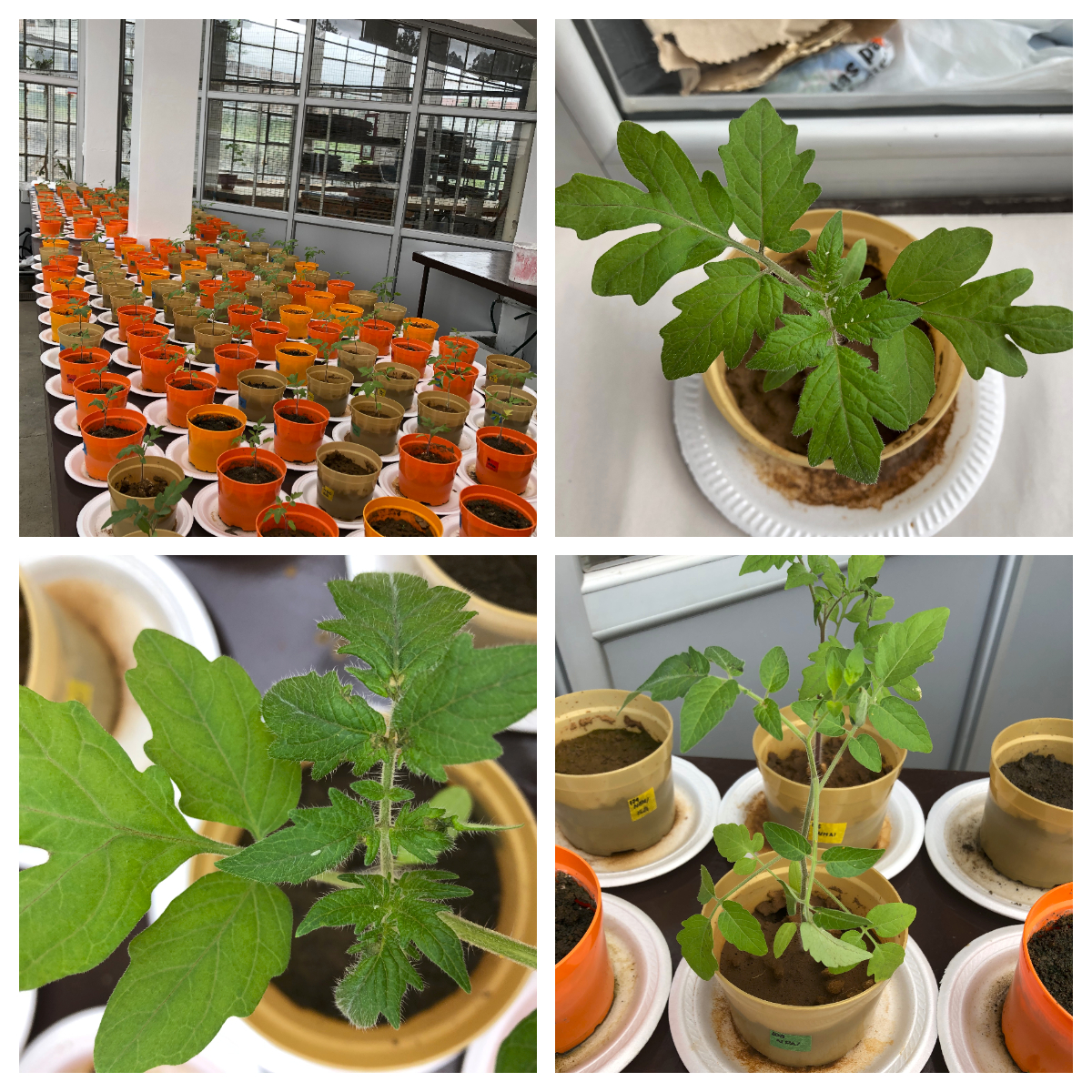


**Figure S1**. Experimental setup for the greenhouse assay conducted in Loja, Ecuador. Tomato plants were grown in different soil treatments and subjected to infestation by Prodiplosis longifila larvae. It is shown the randomized pot arrangement and general growth conditions inside the greenhouse. Plant were monitored over a 7-day period, targeting the L2 larval stage responsible for the most intense herbivory.


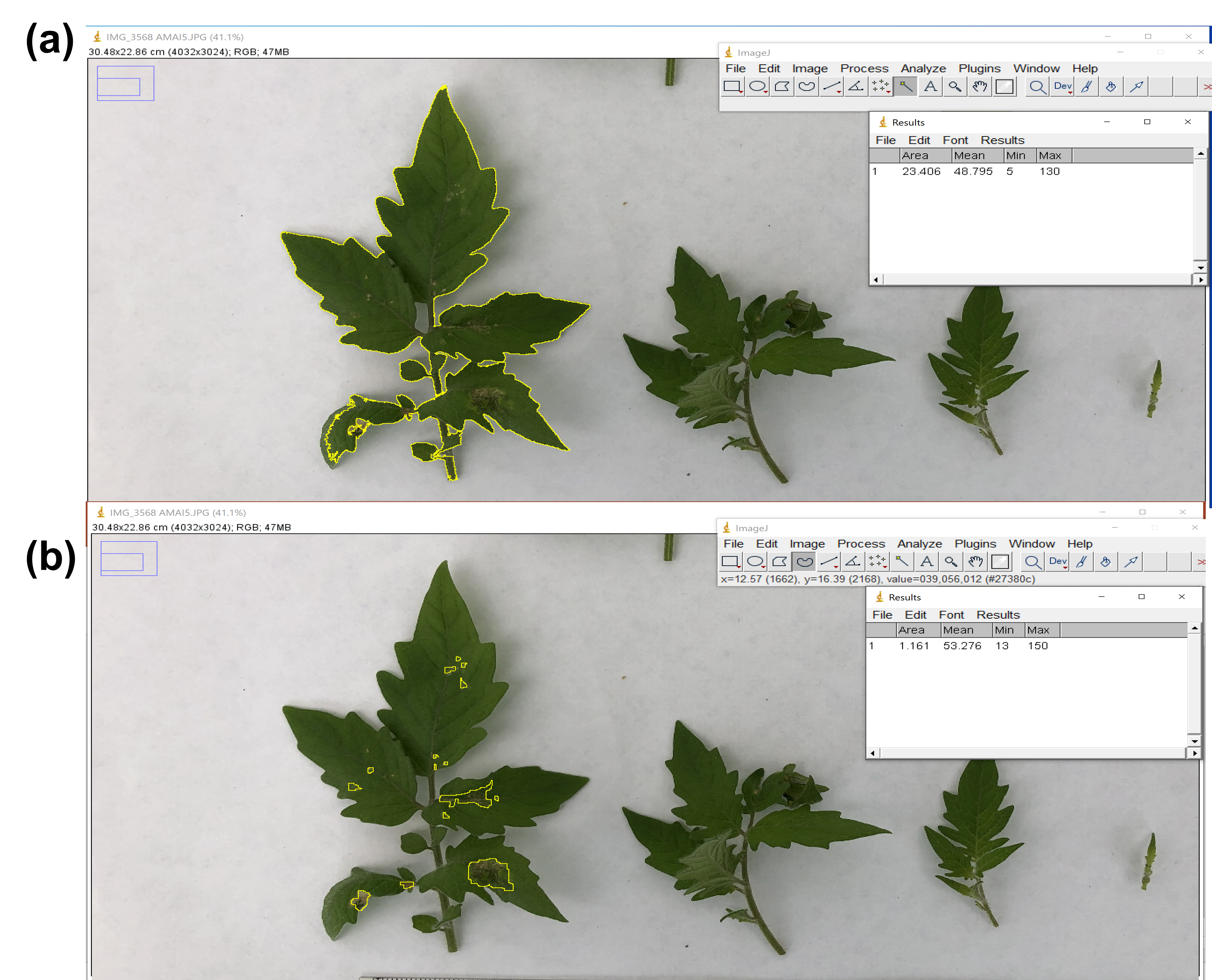


**Figure S2**. Illustration of leaf damage assessment using the ImageJ software from the greenhouse experiment conducted in Loja, Ecuador. Tomato leaves were collected from plants infested with Prodiplosis longifila larvae. Top panel **(a)** shows the measurement of total area per leaf, while the bottom panel **(b)** highlights the are affected by necrosis areas caused by larval feeding. Yellow outlines represent the regions selected for pixel-based quantification, from which the proportion of leaf damage area was calculated for each leaf.


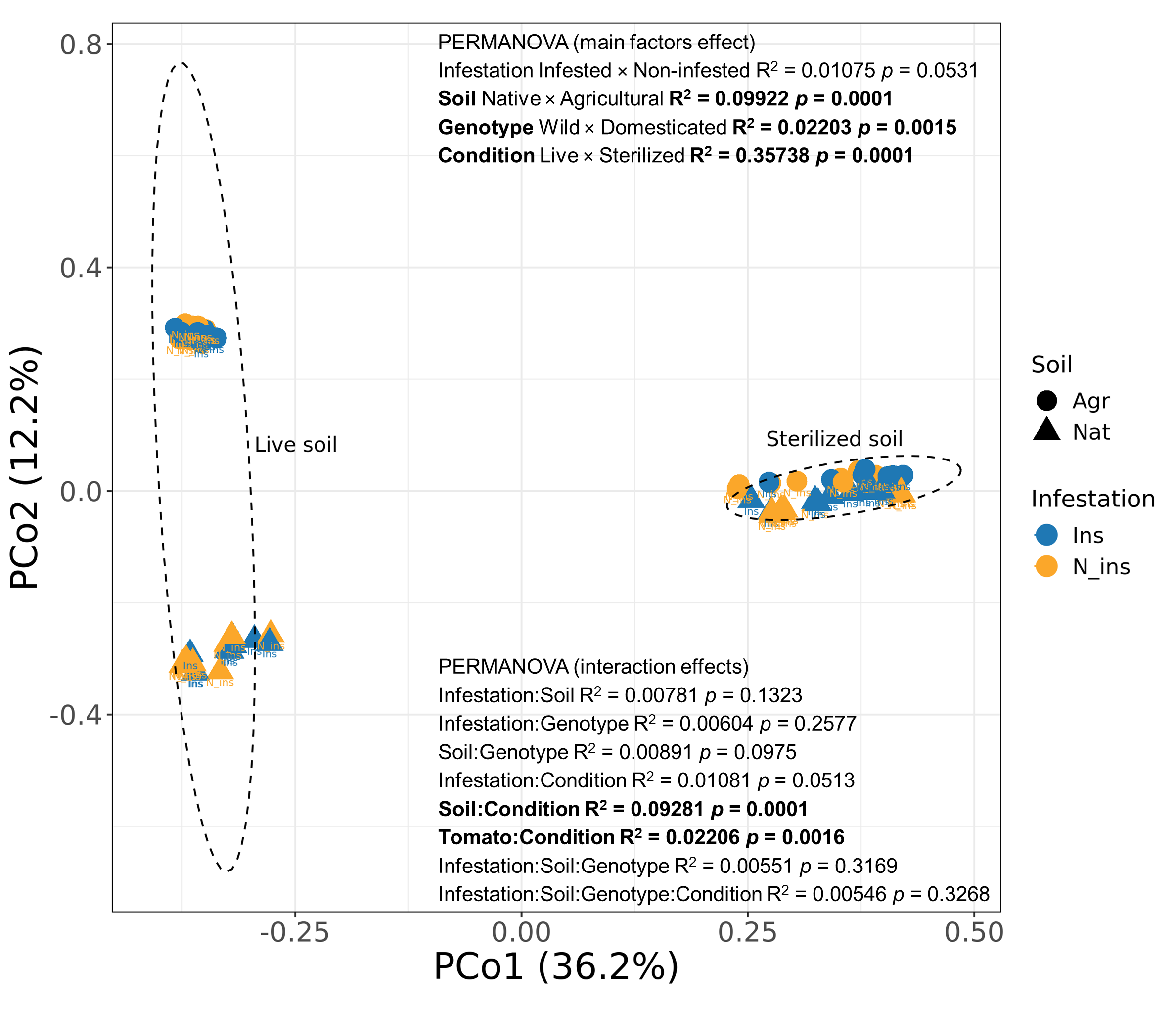


**Figure S3.** PCoA of bacterial communities in the rhizosphere of domesticated S. lycopersicum cv. Moneymaker and wild S. pimpinellifolium tomato. The analysis contrasts infested (Ins) and non-infested (N_ins) plants by Prodiplosis longifila considering different soil types (Agr: Agricultural vs. Nat: Native) and soil conditions (Live and Sterilized).


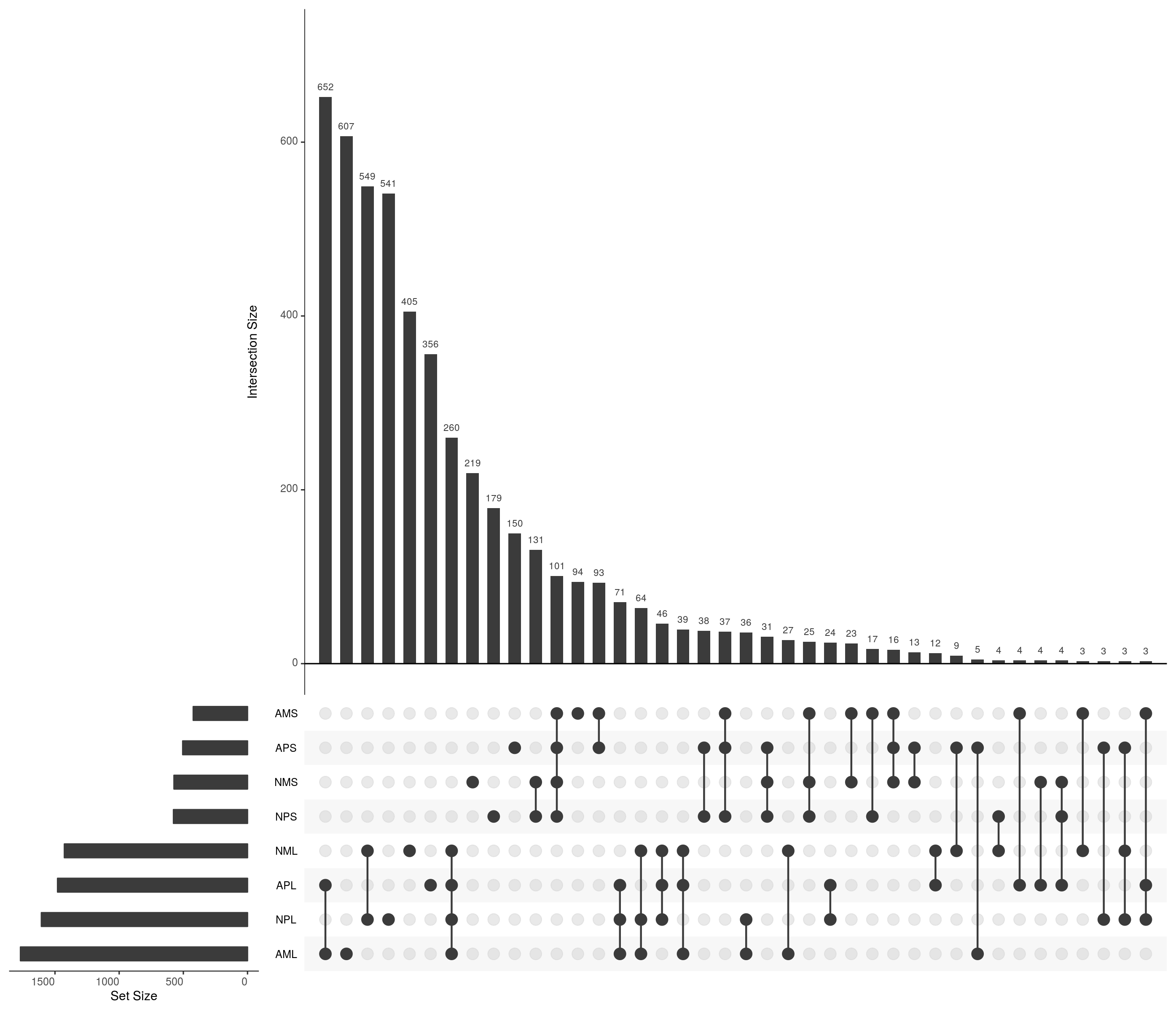


**Figure S4.** Number of bacterial ASVs shared among live and sterilized soils cultivated with wild S. pimpinellifolium and domesticated tomato S. lycopersicum var. Moneymaker. Code samples with: A: Agricultural, N: Native soil; M: S. lycopersicum var. Moneymaker, P: S. pimpinellifolium tomato; L: Live and S: Sterilized soil. Upset plot generated by UpSetR software https://gehlenborglab.shinyapps.io/upsetr/ (Lex et al., 2014).

**
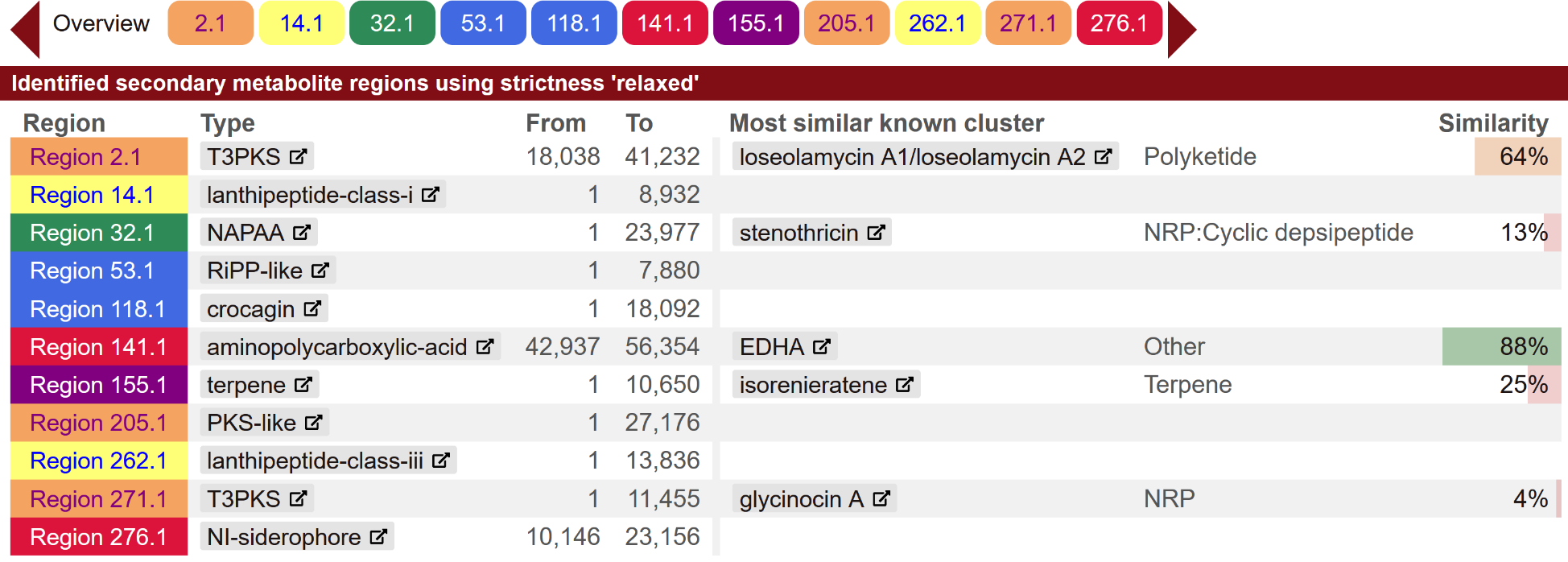
**

**Figure S5**. Overview of BGCs found in Micromonosporaceae MAG Actinoplanes bin 580 by bacterial antiSMASH software (Blin et al., 2023).
